# Psychosis secondary to COVID‐19 in pediatric sickle cell disease

**DOI:** 10.1002/ccr3.8536

**Published:** 2024-02-13

**Authors:** Ashish Khanchandani, Chinonso Onuoha, Beng Fuh

**Affiliations:** ^1^ Brody School of Medicine at East Carolina University Greenville North Carolina USA; ^2^ Department of Pediatrics Brody School of Medicine at East Carolina University Greenville North Carolina USA; ^3^ ECU Health Medical Center Greenville North Carolina USA

**Keywords:** COVID‐19, pediatrics, psychosis, sickle cell disease

## Abstract

**Key Clinical Message:**

COVID‐19 psychosis is a potential long‐term sequela of COVID‐19. Vulnerable populations, such as individuals with sickle cell disease, are at high risk for psychosis. Given the limited number of cases, more investigations in the etiopathology and management of this new disease is needed.

**Abstract:**

We report a case of a 15‐year‐old female with a past medical history of depression who developed psychosis post‐SARS‐CoV‐2 infection (COVID‐19). After an initial moderate COVID‐19 infection, the patient appeared to recover and was discharged home. Four weeks later, she presented with symptoms of psychosis and symptoms of cognitive impairment. Imaging studies did not show any evidence of stroke and toxicology studies were negative. She was treated with antipsychotics and required inpatient neuropsychiatric rehabilitation. Acute psychotic syndrome resolved after 3 weeks, antipsychotics were weaned, and an antidepressant was initiated. Mild cognitive impairment with significant memory loss persisted for about 1 year. Thereafter, she returned to her baseline but remains on an antidepressant. Some studies have previously reported the occurrence of psychosis in individuals with COVID‐19. This report is the first outline of severe prolonged post‐COVID‐19 psychosis in a child with sickle cell disease. Given the neurologic vulnerability of children with sickle cell disease, these individuals should be monitored for neuropsychiatric symptoms post COVID‐19.

## 1 INTRODUCTION

Though reported to be rare, psychosis has been reported in some patients following COVID‐19 around the world.^1^, ^2^ Given the high incidence of COVID‐19 in recent years, the occurrence may be coincidental or due to an underlying relationship. Given the neurologic complications of COVID‐19 including symptoms of multisystem inflammatory syndrome in children (MIS‐C), psychotic complications are not surprising.

Psychosis is a mental disorder that impairs the individual's sense of reality. Symptoms may include hallucinations, delusions, incoherent speech, and agitation. During a psychotic episode, the individual may also experience depression, anxiety, insomnia, cognitive impairment, and social withdrawal.^3^

Psychosis in children is most often associated with schizophrenia. This conclusion, however, must be a diagnosis of exclusion. A variety of other conditions can cause symptoms like psychosis. These conditions include substance use disorder, encephalopathy, immunologic disorders, brain tumor, delirium, sepsis, bacterial disease, hyperthyroidism, Wilson's disease, and epilepsy.^3^ Some individuals are more prone to develop psychosis due to a combination of inherited, biological, psychological, and environmental factors.

There is a growing body of literature suggesting that COVID‐19 can be associated with psychosis. These reports are mostly adult patients, and most cannot definitively conclude a causal relationship. Additionally, large‐scale epidemiological data demonstrated a clear association between the risk of developing psychotic disorders and previous hospitalization for severe infection, and presence of autoimmune diseases such as systemic lupus erythematosus. The risk is often greatest soon after diagnosis of infection. Cytomegalovirus (CMV), herpes simplex virus (HSV), Epstein–Barr Virus (EBV), Influenza, and Toxoplasma gondii have been linked to childhood psychotic experiences.^4^ The possibility that psychosis could occur as a manifestation of COVID‐19 infection is not surprising given the severe immunologic response during COVID‐19 and MIS‐C.

Sickle cell disease is an inherited autosomal recessive blood disorder resulting from a variant of the beta globin gene. Red blood cells of individuals with sickle cell disease polymerize in certain conditions leading to sickling. Complications of sickle cell disease can involve all organs and systems. One of the most devastating complications of sickle cell disease is stroke.^5^ During the disease course, 10% of individuals with sickle cell disease would develop over stroke by age 16 years and 33% would develop silent infarct by age 15 years.^3^, ^5^ In addition to the overt deficits these conditions can cause, sickle cell disease also causes other subtle neurocognitive impairments. Patients with sickle cell disease have a baseline elevated inflammatory state and are mild to moderately immunocompromised. There has, however, been no link between sickle cell disease and psychotic symptoms.

The combination of risk of brain injury heightened inflammatory state, and compromised immune systems make individuals with sickle cell disease more vulnerable to SARS‐CoV‐2 infection, COVID‐19, and the complications within the neuropsychiatric system. More research to definitively link or de‐link COVID‐19 and sickle cell and psychosis is needed.

## 2 CASE REPORT

A 15‐year‐old female with sickle cell disease, HbSS, and history of depression and constipation presented to the emergency department in November of 2020 with complaints of shortness of breath and chest, leg, and back pain. She was afebrile and hypoxic with oxygen saturation being 100% on room air. Chest X‐ray showed left basilar opacity. She was started on 2 L of supplemental oxygen via nasal cannula, analgesics, and antibiotics and admitted for further management. On admission, morphine via patient‐controlled analgesia (PCA) was started and she received a packed red blood cell (pRBC) transfusion. On hospital Day 2, her shortness of breath worsened, and she tested positive for SARS‐CoV‐2 “COVID‐19” via polymerase chain reaction (PCR). SARS‐CoV‐2 IgG was negative. C‐reactive protein (CRP) was elevated at 252.6 mg/dL (0.1–1.7), procalcitonin was mildly elevated at 0.72 ng/mL (0.0–0.5). The platelet count was low at 38 K/μL and the white blood count and differential were normal. Computed chest angiogram (CTA) of the chest and computed tomography (CT) of the abdomen demonstrated ground glass pulmonary opacities L>R with no evidence of pulmonary embolism (PE). Due to concerns for acute decompensation in respiratory status in the setting of COVID‐19 pneumonia with increased work of breathing, she was transferred to the pediatric intensive care unit (PICU) and received high‐dose corticosteroids, remdesivir, enoxaparin, ceftriaxone, and a platelet transfusion. She clinically improved and was transferred to the regular unit the next day. She completely recovered by hospital Day 7 and was discharged home. Inflammatory markers were down‐trending at discharge with CRP and procalcitonin at 98.6 mm/h and 0.15 ng/mL, respectively. At discharge, anticoagulation therapy was switched from enoxaparin to apixaban. She continued her home medication that included oral contraception, folic acid, hydroxyurea, and pain medications as needed.

Twenty days after discharge, the patient experienced an acute psychotic episode. The patient was brought back to the emergency department for concerns of “odd behavior since the morning.” For context, our patient has a pertinent psychiatric history of depression and had a prior trial of an antidepressant and therapy prior to her COVID‐19 infection. In the emergency department, the patient was noted to be scared, jumpy to slight movements, and stating odd things. She mentioned her best friend “Claire” and then acknowledged that she does not know who “Claire” is. Furthermore, her family knows of no friend named Claire. An emergency department physician commented that the patient was GCS 15, startled easily, is confused, and is in an anxious mood. The workup in the Emergency Department found everything normal including a normal procalcitonin of 0.02, mildly elevated CRP of 4.2 mg/dL and an EKG showing sinus tachycardia. She was admitted to the PICU and a pRBC exchange transfusion was performed due to mental status changes in the setting of sickle cell disease and the concern of stroke.

The mother (biological aunt and guardian) had numerous concerns for the patient's mental status. She stated that the patient had been hearing voices telling her to “give up” and that the patient will converse with people who are not present in the room. The patient refused her mother's attempt to reconnect and has been questioning her own sexuality. The patient also had been casting spells in her bedroom to protect her family. The mother was concerned that the patient may have ingested something that may have caused her to hallucinate given that she has access to essential oils and some spices were missing from the kitchen. Furthermore, the patient would occasionally sit in her room in darkness, stay up without sleep, make strange faces, and talk to the mirror.

Infectious and toxicology evaluations were negative (normal). Neurology was also consulted, and the EEG was normal. Magnetic resonance tomography/magnetic resonance angiography (MRI/MRA) and lumbar puncture (LP) reported normal findings and a psychiatry consultation was ordered. The psychiatrist evaluation noted that the “patient is a poor historian.” The psychiatrist further noted that the patient endorsed auditory hallucinations that were intermittently present. The patient believed the voices were real but could not affirm when the voices began. The patient has never used tobacco, alcohol, or illicit substances but had swallowed pills her friends gave her. Her mother stated that these occurrences would not have been possible since the patient had not seen any friends in recent months due to COVID‐19 restrictions. The patient stated that she had no friends and spent a lot of time on her computer, to which she had given the name “Claire.” The patient denied any somatic complaints and was aware of person, place, and time. The patient was prescribed trazodone 50 mg QHS for sleep and Risperdal 0.5 mg QHS and Risperdal 0.25 QAM for psychosis. The psychiatrist further recommended delirium precautions and a sitter in her room.

The patient was discharged to inpatient rehabilitation due to unsteady gait and poor speech. Five days later, she was discharged home. Risperidone was discontinued at discharge. In January 2021, she was seen by PCP and started on escitalopram 10 mg daily. Trazadone was stopped due to the patient feeling “spacey.” In March 2021, the patient admitted to the return of the auditory hallucinations during a hematology/oncology sickle cell follow‐up. She had not been able to resume school and had not established with an outpatient psychiatrist. She established care with a psychologist.

In the next several months, she continued to improve steadily and by December 2021, all symptoms had resolved, and she was back to her pre‐November 2020 baseline. Memory deficits and extreme fearfulness were the last symptoms to resolve. Most recent brain imaging including MRI/MRA (on 10/29/2021) and Transcranial doppler (on 4/28/2022) were normal.

The patient's past medical history may bring some of these details into context. She has sickle cell disease, constipation, and was diagnosed with depression in April 2019. In April 2019, she was evaluated by PCP for being more “withdrawn” after the aunt died several months prior. She was started on escitalopram 10 mg daily. The patient is an exceptional student, earning straight A's. In a follow‐up PCP visit on 7/2019, she had stopped taking escitalopram due to “not feeling right on it” and she was described as happy during that visit and seeing a counselor since April 2019.

## 3 DISCUSSION

The etiology and related pathophysiology associated with COVID‐19 psychosis remain unclear. The etiology of psychotic symptoms may be related to the overall stress of the COVID‐19 pandemic, especially in a psychiatrically vulnerable individual like our patient but preoccupation of COVID‐19 makes this etiology less likely as a sole cause of her psychosis. Additionally, stress after an intensive care unit (ICU) admission can also be a contributory factor.

Corticosteroid‐induced psychosis is a phenomenon which is a diagnosis of exclusion that has been reported in the past. This phenomenon commonly occurs after stopping high doses of oral steroid or an IV equivalent. Our patient who developed psychotic symptoms weeks after the steroid medication received a short course of IV solumedrol for 4 days, making high‐dose corticosteroids another possible but an unlikely etiology.

Sickle cell disease affects the brain and immune systems in numerous ways. Even individual without radiographic or clinical evidence of stroke may have brain or central nervous system blood vessel injury. The inflammatory and circulatory changes resulting from COVID‐19 may lead to neuropsychiatric complications. High doses of corticosteroids are usually used with caution or avoided in individuals with sickle cell disease due to their risk of inducing Vaso‐occlusive pain events. As stated above, high doses of corticosteroids can cause psychosis. Steroid use in patients with vulnerable brain tissue may decrease the threshold of psychosis. The time‐lapse between their use in this patient and the development of psychotic symptoms makes this cause an unlikely etiology.

Our patient has a history of depression and had previously been on antidepressant and counseling therapy prior to her COVID‐19. However, the onset of her depression is clearly related to the death of her aunt which is not an unreasonable response. In addition, her depression had responded well to treatment. The likelihood that this experience, the prevailing pandemic environment, chronic illness, and recent hospitalization made her psychiatrically vulnerable is quite high.^5^

Other postulated etiologies implicated are immune‐based triggers that occur through direct viral infiltration of the CNS and cytokine activation that produce neuropsychiatric symptoms. CRP which was found to be elevated in our patient has been studied to be a potential peripheral marker of immune activation. Other viruses that have been known to produce similar neuropsychiatric symptoms as COVID‐19 are Influenza, H1N1, encephalitis lethargica, and SARS‐CoV‐1 and among other viruses.

There is a growing body of evidence that the immunologic response during COVID‐19 causes varying degrees of neuropsychiatric complications. The “long covid” syndrome sometimes includes neurologic and psychiatric symptoms. The long duration of the recovery phase of our patient makes such a mechanism likely.

COVID‐19 psychosis is a complication of COVID‐19 especially in neurologically and psychiatrically vulnerable populations such as individuals with sickle cell disease. This condition is rare, and the disease course may be different in these individuals than those who are not as vulnerable. Given the limited number of cases, more investigations in the etiopathology and management of this new disease is needed. Databases tracking individuals with sickle cell disease and COVID‐19 should include psychosis.

## AUTHOR CONTRIBUTIONS

**Ashish Khanchandani:** Conceptualization; investigation; methodology; writing – original draft; writing – review and editing. **Chinonso Onuoha:** Conceptualization; data curation; formal analysis; investigation; methodology; writing – original draft; writing – review and editing. **Beng Fuh:** Conceptualization; data curation; formal analysis; investigation; methodology; project administration; writing – original draft; writing – review and editing.

## FUNDING INFORMATION

No specific funding was received to support this work.

## CONFLICT OF INTEREST STATEMENT

All authors on this manuscript declare no actual or potential conflicts of interest.

## CONSENT

Written informed consent was obtained from the patient to publish this report in accordance with the journal's patient consent policy.

## Data Availability

Data sharing is not applicable to this article as no new data were created or analyzed in this study.
